# Prophylactic catheterization of uterine arteries with temporary blood flow occlusion in patients at high risk of pospartum hemorrhage: is it a safe technique?

**DOI:** 10.1590/1677-5449.180134

**Published:** 2019-06-26

**Authors:** Alexandre Malta Brandão, Selma Regina de Oliveira Raymundo, Daniel Gustavo Miquelin, André Rodrigo Miquelin, Fernando Reis, Gabriela Leopoldino da Silva, Heloisa Aparecida Galão, Maria Lucia Luiz Barcelos Veloso

**Affiliations:** 1 Faculdade Regional de Medicina de São José do Rio Preto – FAMERP, Departamento de Cardiologia e Cirurgia Cardiovascular e Hospital de Base, São José do Rio Preto, SP, Brasil.; 2 Hospital Austa, São José do Rio Preto, SP, Brasil.; 3 Faculdade Regional de Medicina de São José do Rio Preto – FAMERP, Hospital da Criança e Maternidade – HCM, Departamento de Ginecologia e Obstetrícia, São José do Rio Preto, SP, Brasil.

**Keywords:** uterus, catheterization, postpartum hemorrhage, placenta accreta

## Abstract

**Background:**

Placenta accreta is an important factor in maternal morbidity and mortality and is responsible for approximately 64% of emergency hysterectomy cases and about 2/3 of cases of puerperal bleeding.

**Objectives:**

To describe a series of cases of prophylactic uterine catheterization performed to prevent significant postpartum bleeding or during caesarean delivery in pregnant women with a previous diagnosis of accretion.

**Methods:**

A retrospective analysis was conducted of medical records of cases of uterine artery catheterization performed during elective or emergency caesarean sections of patients at high risk of postpartum bleeding.

**Results:**

The catheterization of uterine arteries procedure was performed in fourteen patients. Mean duration of surgery and hospital stay were 214.64 minutes (± 42.16) and 7 days, respectively. All patients underwent obstetric hysterectomy. No patient required embolization. There was no bleeding or need to revisit any patient and there were no complications related to puncture. There was one fetal death and no maternal deaths.

**Conclusions:**

In this study, prophylactic uterine artery catheterization with temporary occlusion of blood flow proved to be a safe technique with low fetal mortality, no maternal mortality, and a low rate of blood transfusion and can be considered an important and effective therapeutic strategy for reduction of maternal morbidity and mortality, especially in pregnant women with anomalous placental attachment. Furthermore, the possibility of uterine preservation with the use of this method is an excellent contribution to therapeutic management of this group of patients. However, randomized clinical trials are needed to evaluate the effectiveness of routine use of the technique.

## INTRODUCTION

Peripartum and postpartum hemorrhage remains one of the most common and most serious complications of delivery and is responsible for almost 25% of maternal deaths, 64% of emergency hysterectomy cases, and 2/3 of cases postpartum bleeding.^1^ It is estimated that approximately 30 to 40% of deaths due to postpartum hemorrhage are avoidable.^2^ The high rates of morbidity and mortality caused by postpartum bleeding have stimulated attempts to find less invasive and more effective techniques that could reduce complications of delivery.^1^^,^ 3 ^,^ 4

The most common causes of hemorrhage are: uterine atony, coagulopathies, uterine rupture, and, especially, disorders of placental attachment (placenta previa or placenta accreta). There has been a significant increase in the incidence of placental abnormalities, caused by the rise in the number of caesarean deliveries, and it is now the greatest cause of postpartum hemorrhage.^5^

Placenta accreta is an incorrect attachment of the placenta to the wall of the uterus, penetrating beyond the endometrium, and there is even a possibility of invasion of adjacent organs. The placenta attaches abnormally to the decidua or to the wall of the uterus. It is believed that this abnormality is the result of a deficiency of decidualization and trophoblast invasion beyond the normal limit, associated with irregularities of the wall of the uterus (scars).^6^^-^ 8 As a result, the chorionic villosities come into direct contact with the myometrium, the blood vessels, or the adjacent tissues.^8^^,^ 9 Risk factors for placenta accreta include prior uterine surgery, maternal age greater than 35 years, multiparity, and submucosal myomas.^6^^,^ 8

Diagnostic suspicion is triggered by supplementary examinations, generally gestational ultrasonography (US). Probable findings in cases of placenta accreta include elimination of the free space between the placenta and the myometrium, visible placental lacunae, and interruption of the bladder-uterus interface.^9^^,^ 10 In doubtful cases, magnetic resonance (MRI) can be helpful.^11^^,^ 12 However, definitive diagnosis can only be arrived at by histopathological examination of the placenta.

At the majority of services, a patient with suspected abnormal placental attachment will be managed with elective caesarean at 34 to 37 weeks’ gestation and hysterectomy.^10^^,^ 13 While preservation of the uterus, with resection of the placenta, is controversial, it can be considered in cases of focal placenta accreta or posterior placenta accreta.^12^^,^ 14 The degree of inflammation and placental invasion are associated with a considerable risk of bleeding, even when a hysterectomy is performed, and with need for blood transfusion.

Prophylactic catheterization of the uterine arteries, with or without embolization, is increasingly being used as a means of avoiding bleeding and its clinical repercussions, and is a supplementary technique that can help to preserve the uterus and reduce bleeding.^6^^-^ 7 ^,^ 13 ^,^ 14

First described in 1979, uterine embolization has been progressively adopted for treatment of postpartum bleeding, and has become recognized as a safe technique, since hysterectomy is not obligatory, preserving the patient’s fertility.^3^^,^ 13 ^,^ 14 It is therefore considered that catheterization of the uterine arteries with temporary occlusion by endovascular balloon is a valid potion in management of patients at high risk of bleeding, in particular those with a preexisting diagnosis of placenta accreta.

Since January 2017, the routine care protocol at the Hospital de Base de São José do Rio Preto has included catheterization of the uterine arteries with inflation of a balloon catheter during elective cesareans when placenta accreta is suspected. This study will therefore evaluate the peripartum management of pregnant women with placental attachment disorders, describing cases of prophylactic catheterization of the uterine arteries during delivery of women with a diagnosis of placenta accreta or previa, demonstrating its efficacy for containment of bleeding after or during the caesarean.

## MATERIALS AND METHODS

This study took place at the Hospital de Base de São José do Rio Preto, Brazil, with retrospective analysis of the medical records from January 2017 to January 2018 of all cases of catheterization of uterine arteries during the peripartum period in patients at high risk of postpartum bleeding. The study complies with the provisions of National Health Council (Conselho Nacional de Saúde) resolution 466 of 12/12/2012, and was submitted to the local Ethics Commission and approved on 28 June 2018 (decision number 2.678.814, CAAE: 88478218.9.0000.5415).

Patients were defined as at high risk of bleeding if they had been diagnosed with or suspected of placenta previa/accreta on the basis of prenatal imaging exams, whether US or MRI. In the majority of cases, the diagnosis of placenta accreta was later confirmed by histopathological examination.

The group investigated comprised patients over the age of 18 years, irrespective of parity, and the variables analyzed were demographic data, intraoperative blood loss volume, complications related to embolization, and length of hospital stay. Fetal vitality was monitored during the procedure by auscultation of fetal heartbeat and the newborns’ Apgar scores were calculated.

The catheterization technique employed consisted of bilateral puncture of the femoral arteries followed by aortoiliac angiography and selective catheterization of the uterine arteries. 5 French cobra catheters on 0.035 hydrophilic guidewires were used to selectively catheterize the uterine arteries. The proximal thirds of the uterine arteries were temporarily occluded with 6 × 40 mm balloon catheters, using inflation syringes, up to the balloons’ nominal pressures, according to their manufacturers’ recommendations. After catheterization and occlusion of the arteries with the balloon catheters, caesarean delivery was performed and the last angiography images were acquired at the end of surgery to analyze bleeding. If bleeding was detected, the pelvic vessels would be embolized with 350– 500µ or 500–700µ microparticles of polyvinyl alcohol **(**PVA), depending on the assessment of the final angiographic images.

Prophylactic catheterization was considered effective if the bleeding was significantly reduced, as demonstrated by the following findings: no need for blood transfusion, for supplementary treatments to achieve hemostasis, or for emergency hysterectomy. The most likely complications would be lower limb ischemia, uterine necrosis, puerperal fever, and poor fetal vitality.

Elective hysterectomy did not affect the assessment of the technical success of uterine catheterization, since the decision was taken prenatally and was purely obstetric.

## RESULTS

From January 2017 to January 2018, 14 uterine catheterization procedures were carried out during the peripartum period. Two (14.2%) of these procedures were performed with emergency cesareans and 12 (85.7%) with elective cesareans. All of these patients had provisional diagnoses of placenta accreta made during prenatal consultations on the basis of imaging exams (US and MRI). Definitive diagnoses were made on the basis of anatomopathological examination of the placenta, confirming placenta accreta in 13 patients (92.8%). In the fourteenth patient, the surgical specimen revealed evidence of placenta percreta and uterine myomatosis. One of the patients who required an emergency caesarean had exhibited reduced fetal movement, resulting in stillbirth due to placental insufficiency, and the other suffered powerful abdominal pains, raising a suspicion of a ruptured uterus, which was not confirmed intraoperatively. There were no maternal deaths and all patients underwent hysterectomy on the basis of obstetric indications.

The mean age of patients was 32.7 years (21 to 40) (± 7.4 years) and mean gestational age in weeks at the time of caesarean was 35.6 weeks (33 to 37) (± 1.4 weeks) (Table 1).

**Table 1:** Characteristics of the women subjected to prophylactic catheterization of the uterine arteries and hysterectomy.

	Minimum	Maximum	Mean	Standard deviation
Age (years)	21	40	32.7	7.4
Gestational age (weeks)	33	37	36	1.4

Catheterization was performed according to the routine protocol at the service, and selective catheterization of the uterine arteries was successful in all of the patients studied. Three patients (21%) required transfusion of packed red blood cells, and one required vasopressor administration during the procedure. All of the patients were transferred to intensive care during the immediate postoperative period, and monitored for episodes of bleeding or hemodynamic instability.

Newborns’ 5-minute Apgar scores ranged from 5 to 9, with a mean of 9. By the 10th minute of life, all newborn infants had an Apgar over 9. In one case, stillbirth was confirmed during the initial approach.

The mean duration of surgery was 214.64 minutes (180 to 300, with a standard deviation of ± 42.16 minutes). Mean duration of elective surgery (n=12) was 218.75 minutes (180 to 300) and mean duration of emergency surgery (n=2) was 190 minutes (180 to 200) (Table 2). Mean length of hospital stay was 7 days, with a standard deviation of ± 3.3 days. There was no difference in duration of surgery between emergency and elective cesareans and none of the patients had renal dysfunction during the postoperative period. There were also no cases of bleeding recurrence and no need for surgical revisits. There were no complications directly related to puncture or embolization of arteries. Control angiography showed that none of the patients required embolization, because there was no residual bleeding.

**Table 2:** Duration of surgery in women who underwent hysterectomy with prophylactic catheterization of the uterine arteries (emergency vs. elective).

**Duration (minutes)**	**Minimum**	**Maximum**	**Mean**	**Standard deviation**	
All operations	180	300	214.64	42.16	p = 0.16
Emergency (n=2)	180	200	190	14.14	
Elective (n=12)	180	300	218.75	44.21	

## DISCUSSION

Postpartum bleeding is one of the most serious obstetric complications, with high rates of morbidity and mortality. In this study, 14 medical records were analyzed from patients diagnosed with placenta accreta who underwent caesareans with prophylactic catheterization of the uterine arteries and balloon catheter inflation followed by hysterectomy.

These procedures were conducted in accordance with the standard protocol at the service and there were no complications related to puncture of the arteries. Lee et al. listed dissection of the uterine arteries, hematoma at the puncture site, paresthesia, and lower limb edema as complications.^15^

All of the patients in our case series underwent hysterectomy because of obstetric indications. This meant that the efficacy of catheterization of the uterine arteries for preservation of patients’ fertility could not be evaluated. Ojala et al.^16^ described a series of cases in which 22 patients underwent embolization of the uterine arteries, in five of whom catheterization was prophylactic. According to Ojala et al., catheterization averted hysterectomy in 80% of cases and the same success rate was reported by Badawy et al.^17^

The mean duration of surgery in the present study was 214,64 minutes, but the time taken for catheterization or its influence on the duration of surgery were not recorded. Omar et al.^18^ and Shrivastava et al.^19^ reported mean durations of 296 and 180 minutes, respectively, for hysterectomy with catheterization, with no statistical difference when compared to hysterectomies performed without the endovascular component.

The reduced uterine blood flow provoked by inflation of the balloon and consequent reduction in uterine blood pressure is responsible for the low prevalence of transfusions, as hypothesized by Kidney et al.^20^ in a 2001 article. In the present study, transfusions were only needed in three patients (21%), and a vasopressor was administered to one patient during the hysterectomy. These data are similar to those from studies by Mitty et al.^21^ and Hansch et al.,^22^ which demonstrated the efficacy of embolization with reduced intraoperative bleeding.

Lee et al.^15^ reported a technical success rate ranging from 85 to 86.2%. The technical success rate in the series described here was 79%. There was one stillbirth during peripartum caused by placental insufficiency and another patient had powerful abdominal pains and a suspected ruptured uterus, but this was not confirmed intraoperatively. There were no maternal deaths.

This study is subject to limitations including the retrospective design, the analysis based on a case series without a control group, and the single-center data. It was also impossible to analyze the impact of embolization of the uterine arteries on patient fertility, since, as mentioned above, all of them underwent hysterectomy because of obstetric indications.

## CONCLUSIONS

In this study, prophylactic catheterization of the uterine arteries with temporary occlusion of blood flow in patients at high risk of postpartum hemorrhage proved to be a safe technique because it was associated with low fetal mortality, a low rate of blood transfusions, and no cases of maternal death.

Prophylactic catheterization of the uterine arteries, with or without embolization, can be considered an important therapeutic strategy that is safe and effective for reducing maternal and fetal morbidity and mortality by controlling blood loss, especially among pregnant women with abnormal placental attachment. Furthermore, the possibility of saving the uterus offered by this method is an excellent contribution to the therapeutic arsenal in this group of patients. However, additional studies with different designs are needed to confirm the efficacy of routine use of this technique.
