# Supplementary material for: Prophylactic catheterization of uterine arteries with temporary blood flow occlusion in patients at high risk of pospartum hemorrhage: is it a safe technique?
Source: J Vasc Bras. 2019 Jun 26;18:e20180134. doi: 10.1590/1677-5449.180134 (PMC6636812; doi:10.1590/1677-5449.180134)
Supplement: Translation

# Translation: Cateterização profilática de artérias uterinas com oclusão temporária do fluxo sanguíneo em pacientes de alto risco para hemorragia puerperal: é uma técnica segura?

**Authors:** Alexandre Malta Brandão, Selma Regina de Oliveira Raymundo, Daniel Gustavo Miquelin, André Rodrigo Miquelin, Fernando Reis, Gabriela Leopoldino da Silva, Heloisa Aparecida Galão, Maria Lucia Luiz Barcelos Veloso

**Affiliations:** 1 Faculdade Regional de Medicina de São José do Rio Preto – FAMERP, Departamento de Cardiologia e Cirurgia Cardiovascular e Hospital de Base, São José do Rio Preto, SP, Brasil.; 2 Hospital Austa, São José do Rio Preto, SP, Brasil.; 3 Faculdade Regional de Medicina de São José do Rio Preto – FAMERP, Hospital da Criança e Maternidade – HCM, Departamento de Ginecologia e Obstetrícia, São José do Rio Preto, SP, Brasil.

**Keywords:** útero, cateterismo, hemorragia pós-parto, placenta acreta

## Abstract

**Contexto:**

A placenta acreta é um importante causa de morbimortalidade materna, sendo responsável por aproximadamente 64% dos casos de histerectomia de urgência e em torno de 2/3 dos casos de sangramento puerperal.

**Objetivos:**

Descrever uma série de casos de cateterização uterina profilática para evitar sangramento significativo no pós-parto ou durante parto cesárea em gestantes com diagnóstico prévio de acretismo.

**Métodos:**

Foi realizada uma análise retrospectiva de prontuários dos casos de cateterização da artéria uterina durante cesarianas eletivas ou de urgência em pacientes com alto risco de sangramento puerperal.

**Resultados:**

O procedimento foi realizado em 14 pacientes. O tempo médio do procedimento cirúrgico e da internação foi de 214,64 minutos (± 42,16) e 7 dias, respectivamente. Todas as pacientes foram submetidas a histerectomia por indicação obstétrica. Nenhuma paciente necessitou de embolização. Não houve sangramento ou necessidade de reabordagem em nenhuma paciente e nenhuma complicação relacionada à punção. Houve apenas um caso de morte fetal e nenhuma morte materna.

**Conclusões:**

Neste estudo, a cateterização profilática de artérias uterinas com oclusão temporária do fluxo sanguíneo demonstrou ser uma técnica segura, pois apresentou baixa mortalidade fetal, baixa necessidade de hemotransfusão, e nenhuma morte materna. Portanto, pode ser considerada uma estratégia terapêutica importante e eficaz para a diminuição da morbimortalidade materna, especialmente em gestantes com implantação placentária anômala. Além disso, a possibilidade de preservação uterina com o uso do método traz excelente contribuição na terapêutica nesse grupo de pacientes. Entretanto, são necessários ensaios clínicos randomizados para avaliar a eficácia do uso rotineiro da técnica.

## INTRODUÇÃO

A hemorragia peri e pós-parto ainda é uma das principais e mais graves complicações puerperais, sendo a causa de quase 25% das mortes maternas, de 64% dos casos de histerectomia de urgência e de 2/3 dos casos de sangramento puerperal^1^. Estima-se que aproximadamente 30 a 40% das mortes decorrentes da hemorragia puerperal poderiam ter sido evitadas^2^. Desse modo, a alta morbimortalidade resultante do sangramento pós-parto levou à busca por técnicas menos invasivas e mais eficazes, capazes de reduzir as complicações puerperais^1^^,^ 3 ^,^ 4.

Entre as principais causas de hemorragia, destacam-se as seguintes: atonia uterina, coagulopatias, ruptura uterina e, especialmente, defeitos na implantação placentária (placenta prévia ou acreta). Houve um aumento significativo na incidência de anormalidades placentárias, em função do crescimento no número de partos cesáreas, transformando-as na principal causa de hemorragia puerperal^5^.

O acretismo placentário consiste na implantação incorreta da placenta na parede uterina, ultrapassando o endométrio, com possibilidade de invasão até de órgãos adjacentes. A placenta adere-se de forma anormal à decídua ou à parede uterina. Acredita-se que essa alteração decorra de uma deficiência na decidualização e de uma invasão trofoblástica além do limite normal, associadas a irregularidades na parede uterina (cicatrizes)^6^^-^ 8. Dessa maneira, ocorre um contato direto das vilosidades coriônicas com o miométrio, os vasos sanguíneos ou os tecidos adjacentes^8^^,^ 9. São fatores de risco da placenta acreta: cirurgia uterina prévia, idade materna maior que 35 anos, multiparidade e miomas submucosos^6^^,^ 8.

A suspeita diagnóstica é baseada em exames complementares, geralmente ultrassonografia (US) gestacional. Os achados prováveis de placenta acreta são: obliteração de espaço livre entre a placenta e o miométrio, visualização de lacunas placentárias e interrupção da interface bexiga-útero^9^^,^ 10. Nos casos incertos, a ressonância magnética (RM) pode ser feita^11^^,^ 12. O diagnóstico definitivo, no entanto, é realizado somente com o exame histopatológico da placenta.

Diante de uma paciente com suspeita de implantação placentária não habitual, a cesariana eletiva entre 34 e 37 semanas gestacionais, associada à histerectomia, é a opção na maioria dos serviços^10^^,^ 13. A preservação uterina com ressecção placentária, embora controversa, pode ser considerada em casos de placenta acreta focal ou placenta acreta posterior^12^^,^ 14. Devido ao grau de inflamação e invasão placentária, existe um grande risco de sangramento, mesmo quando a histerectomia é realizada, havendo necessidade de hemotransfusão.

Como forma de evitar o sangramento e suas repercussões clínicas, a cateterização profilática das artérias uterinas, com ou sem embolização, vem sendo cada vez mais empregada, constituindo medida auxiliar na preservação uterina ou na diminuição do sangramento^6^^-^ 7 ^,^ 13 ^,^ 14.

Descrita primeiramente em 1979, a embolização uterina tem sido progressivamente adotada no tratamento do sangramento pós-parto, destacando-se como uma técnica segura, visto que a histerectomia não é obrigatória, preservando a fertilidade da mulher^3^^,^ 13 ^,^ 14. Assim, estima-se que a cateterização das artérias uterinas e sua oclusão temporária por meio de balão endovascular tenha sua validade na propedêutica de pacientes de alto risco para sangramento, em particular daquelas com diagnóstico prévio de acretismo placentário.

Desde janeiro de 2017, tornou-se protocolo assistencial, no Hospital de Base de São José do Rio Preto, a cateterização das artérias uterinas com insuflação de cateter-balão durante a realização de cesarianas eletivas quando havia suspeita de acretismo placentário. Este trabalho propõe, então, a avaliação da propedêutica periparto em gestantes com defeitos na implantação placentária, descrevendo os casos de cateterização profilática das artérias uterinas durante o parto de gestantes com diagnóstico prévio de acretismo ou placenta prévia e demonstrando sua eficácia na contenção do sangramento pós-parto ou durante a cesariana.

## MATERIAL E MÉTODOS

O presente estudo teve lugar no Hospital de Base de São José do Rio Preto, com análise retrospectiva de prontuários de janeiro de 2017 a janeiro de 2018 de todos os casos de cateterização das artérias uterinas no período periparto em pacientes de alto risco para sangramento puerperal. Além disso, está de acordo com a resolução do Conselho Nacional de Saúde nº 466 de 12/12/2012, tendo sido submetido ao Conselho de Ética e Pesquisa e aprovado em 28/06/18 (número do parecer: 2.678.814, CAAE: 88478218.9.0000.5415).

Considerou-se alto risco para sangramento como diagnóstico ou suspeita de placenta prévia/acreta em exame de imagem no período pré-natal, por US ou RM. O diagnóstico de acretismo placentário confirmou-se pelo exame histopatológico na maioria dos casos.

O grupo investigado foi constituído de pacientes maiores de 18 anos, independente da paridade, e foram analisados seus dados demográficos, volume perdido de sangue no intraoperatório, complicações decorrentes da embolização, e tempo de internação. Avaliou-se a vitalidade fetal durante o procedimento por meio da ausculta dos batimentos cardiofetais, sendo também verificado o índice de Apgar do recém-nascido.

A técnica de cateterização consistiu na punção bilateral das artérias femorais seguida de angiografia aortoilíaca e cateterização seletiva das artérias uterinas. Utilizaram-se cateteres cobra 5F guiados por fio-guia hidrofílico 0,035 para seletivar as artérias uterinas. As artérias uterinas foram ocluídas momentaneamente em seu terço proximal com cateteres-balões 6 × 40mm com auxílio de seringa insufladora até a pressão nominal do balão, de acordo com sua marca. Após a cateterização e a oclusão das artérias pelos cateteres-balões, realizou-se a cesariana, e uma última imagem de angiografia foi obtida no término da cirurgia para análise do sangramento. Se ele fosse detectado, seria realizada a embolização dos vasos pélvicos com micropartículas de álcool polivinílico **(**PVA) de 350– 500µ ou 500–700µ, dependendo da avaliação angiográfica final.

A cateterização profilática foi considerada eficaz se houvesse diminuição significativa do sangramento, demonstrada pelos seguintes achados: ausência de hemotransfusão, de tratamento complementar para hemostasia ou de histerectomia de urgência. Prováveis complicações seriam isquemia do membro inferior, necrose uterina, febre puerperal e baixa vitalidade fetal.

A histerectomia eletiva não influenciou na avaliação do sucesso técnico da cateterização uterina, pois se tratou de indicação pré-natal e exclusivamente obstétrica.

## RESULTADOS

De janeiro de 2017 a janeiro de 2018, efetuaram-se 14 cateterizações uterinas no período periparto. Dessas, duas (14,2%) ocorreram em cesarianas de urgência e 12 (85,7%) em cesarianas eletivas. Todas as pacientes apresentavam hipótese diagnóstica de placenta acreta nos exames de imagem (US e RM) nas consultas de pré-natal. Obteve-se o diagnóstico definitivo por meio de exame anatomopatológico da placenta, sendo confirmado acretismo placentário em 13 pacientes (92,8%). Em uma paciente a peça cirúrgica evidenciou placenta percreta associada a miomatose uterina. Entre as pacientes que precisaram de cesariana de urgência, uma apresentou diminuição da movimentação fetal, evoluindo com morte fetal no periparto devido a insuficiência placentária, e a outra teve cólicas abdominais de forte intensidade com suspeita de rotura uterina, que não se confirmou no intraoperatório. Não houve casos de morte materna e todas as pacientes foram submetidas a histerectomia por indicação obstétrica.

A idade média das pacientes foi de 32,7 anos (21 a 40) (± 7,4 anos), e a idade gestacional média, em semanas, no momento da cesariana era de 35,6 semanas (33 a 37) (± 1,4 semanas) (Tabela 1).

**Tabela 1:** Características das gestantes submetidas a cateterização profilática das artérias uterinas e histerectomia.

	Mínimo	Máximo	Média	Desvio padrão
Idade (anos)	21	40	32,7	7,4
Idade gestacional (semanas)	33	37	36	1,4

A cateterização atendeu ao protocolo do serviço, obtendo-se sucesso na seleção das artérias uterinas em todas as pacientes do estudo. Necessitou-se de transfusão de concentrado de hemácias em três pacientes (21%), sendo que uma delas precisou de vasopressor durante o procedimento. Todas as pacientes permaneceram em terapia intensiva durante o pós-operatório imediato, com monitorização quanto a episódios de sangramento ou de instabilização hemodinâmica.

O Apgar do recém-nascido no 5° minuto de vida variou de 5 a 9, com uma média de 9. No 10º minuto de vida, todos os recém-nascidos apresentaram Apgar acima de 9. Somente em um caso atestou-se morte fetal durante o atendimento primário.

O tempo médio de procedimento cirúrgico foi de 214,64 minutos (180 a 300, com desvio padrão de ± 42,16 minutos). Nas cirurgias eletivas (12), esse tempo foi de 218,75 minutos (180 a 300), e nas cirurgias de urgência (2), de 190 minutos (180 a 200) (Tabela 2). O tempo médio de internação foi de 7 dias, com desvio padrão de ± 3,3 dias. Não houve diferença no tempo cirúrgico ao se comparar as cesarianas de urgência e eletivas, e nenhuma das pacientes manifestou alteração da função renal no período pós-operatório, não havendo também ressangramento ou necessidade de reabordagem. Não se observou qualquer complicação diretamente relacionada à punção ou à embolização arterial. Após a angiografia de controle, constatou-se que nenhuma paciente necessitava de embolização, pois não havia sangramento remanescente.

**Tabela 2:** Tempo cirúrgico das gestantes submetidas a histerectomia e cateterização profilática das artérias uterinas (urgência × eletiva).

**Tempo (minutos)**	**Mínimo**	**Máximo**	**Média**	**Desvio padrão**	
Todas as cirurgias	180	300	214,64	42,16	p = 0,16
Urgência (n=2)	180	200	190	14,14	
Eletiva (n=12)	180	300	218,75	44,21	

## DISCUSSÃO

O sangramento puerperal é uma das mais temidas complicações obstétricas, com grande morbimortalidade. Neste estudo, analisaram-se 14 prontuários de pacientes com diagnóstico de acretismo placentário submetidas à cesariana, associada a cateterização profilática de artérias uterinas com insuflação de cateter-balão seguida de histerectomia.

O procedimento foi feito de acordo com protocolo do serviço, não havendo complicações nas punções arteriais. Dissecção das artérias uterinas, hematoma no local da punção, parestesia e edema dos membros inferiores são complicações descritas no estudo de Lee et al.^15^.

Em nossa série de casos, todas as pacientes submeteram-se a histerectomia por indicação obstétrica. Dessa forma, a eficácia da cateterização das artérias uterinas na manutenção da fertilidade da gestante não foi possível de ser avaliada. Ojala et al.^16^, em sua série de casos, observaram 22 pacientes submetidas a embolização uterina, sendo que, em cinco delas, as cateterizações foram profiláticas. A cateterização, segundo Ojala et al., evitou a histerectomia em 80% dos casos, mesma taxa de sucesso constatada no estudo de Badawy et al.^17^.

O tempo cirúrgico médio no presente estudo foi de 214,64 minutos, não se averiguando o tempo necessário para a cateterização e sua influência no tempo cirúrgico. Omar et al.^18^ e Shrivastava et al.^19^ detectaram um tempo médio de 296 e 180 minutos, respectivamente, na histerectomia com cateterização, porém sem diferença estatística quando comparada à histerectomia sem a abordagem endovascular.

A diminuição do fluxo sanguíneo uterino pela insuflação do balão, com consequente redução da pressão sanguínea uterina, teorizada por Kidney et al.^20^ em seu artigo de 2001, justifica a baixa prevalência de transfusões. No presente estudo, também só foram necessárias transfusões em três pacientes (21%), sendo usado o vasopressor em uma delas durante a histerectomia. Esses dados assemelham-se aos dos estudos de Mitty et al.^21^ e Hansch et al.^22^, que demonstraram eficácia na embolização, com diminuição do sangramento no intraoperatório.

Lee et al.^15^ relataram uma taxa de sucesso técnico que variou de 85 a 86,2%. A taxa de sucesso técnico na série aqui descrita foi de 79%. Ocorreu apenas uma morte fetal no periparto devido a insuficiência placentária e a outra teve cólicas abdominais de forte intensidade com suspeita de rotura uterina, porém não confirmada no intraoperatório. Não houve casos de morte materna.

O presente estudo apresentou como limitações o fato de ter um desenho retrospectivo, avaliar uma série de casos sem grupo controle e ser unicêntrico. Além disso, não propiciou a análise do impacto da embolização uterina na fertilidade das pacientes, uma vez que, conforme já dito, todas foram submetidas a histerectomia por indicação obstétrica.

## CONCLUSÕES

Neste estudo, a cateterização profilática de artérias uterinas com oclusão temporária do fluxo sanguíneo em pacientes de alto risco para hemorragia puerperal demonstrou ser uma técnica segura porque apresentou baixa mortalidade fetal, baixa necessidade de hemotransfusão, e nenhum caso de mortalidade materna.

A cateterização profilática das artérias uterinas, com ou sem embolização, pode ser vista como uma estratégia terapêutica importante, segura e eficaz para a diminuição da morbimortalidade materno-fetal, especialmente em gestantes com implantação placentária anômala, por meio do controle da perda sanguínea. Além disso, a possibilidade de preservação uterina com o uso do método traz excelente contribuição ao arsenal terapêutico nesse grupo de pacientes. Entretanto, são necessários outros estudos com desenhos diferentes para comprovar a eficácia do uso rotineiro da técnica.
